# 5-hydroxymethylcytosine loss is associated with poor prognosis for patients with WHO grade II diffuse astrocytomas

**DOI:** 10.1038/srep20882

**Published:** 2016-02-11

**Authors:** Feng Zhang, Yifan Liu, Zhiwen Zhang, Jie Li, Yi Wan, Liying Zhang, Yangmei Wang, Xia Li, Yuqiao Xu, Xin Fu, Xiumin Zhang, Ming Zhang, Zhekai Zhang, Jing Zhang, Qingguo Yan, Jing Ye, Zhe Wang, Charlie Degui Chen, Wei Lin, Qing Li

**Affiliations:** 1 State Key Laboratory of Cancer Biology, Department of Pathology; Xijing Hospital, Fourth Military Medical University, Shaanxi, 710032, China; 2 State Key Laboratory of Molecular Biology, Shanghai Key laboratory of Molecular Andrology, Institute of Biochemistry and Cell Biology, Shanghai Institutes for Biological Sciences, Chinese Academy of Sciences, 320 Yueyang Road, Shanghai 200031, China; 3 Department of Health Statistics, Fourth Military Medical University, Shaanxi, 710032, China; 4 Company 13, Student Brigade, Fourth Military Medical University, Xi’an, 710032, China; 5 Department of Neurosurgery; Xijing Hospital, Fourth Military Medical University, Shaanxi, 710032, China

## Abstract

Currently, the reliable prognostic biomarkers for WHO grade II diffuse astrocytomas (DA) are still limited. We investigated the relations between the level of 5-Hydroxymethylcytosine (5hmC), an oxidated production of 5-methylcytosine (5mC) by the ten eleven translocated (TET) enzymes, and clinicopathological features of glioma patients. With an identified anti-5hmC antibody, we performed immunohistochemistry in 287 glioma cases. We detected that 5hmC variably reduced in most gliomas and 5hmC reduction was closely associated with higher pathological grades and shortened survival of glioma patients. In multivariate analysis, 5hmC had no independent prognostic value in the entire patient cohort. However, multivariate analysis within subtypes of gliomas revealed that 5hmC was still a prognostic marker confined to DA. In addition, we detected that *IDH1* mutation by DNA sequencing was associated with favorable survival within DA. Lastly, we detected that the combination of 5hmC/KI67 was a useful prognostic marker for restratification of DA.

Human gliomas are a heterogeneous group of tumors and traditionally classified into various subtypes and grades mostly based on their microscopic characteristics for therapeutic decision-making^1^,^2^. However, histopathological criteria usually unavoidablely cause subjectively diagnostic interobserver variability^3^,^4^. Moveover, classification based on microscopic characteristics rather than molecular pathogenesis of gliomas limits the adequate assessment of prognosis and appropriate planning of treatment. For these regards, “ISN-Haarlem” guidelines recently proposed to define diagnostic entities as narrowly as possible and to include applicable molecular data to come up with a more objective and reproducible “integrated diagnosis” for glioma classification^5^. For example, molecular biomarkers isocitrate dehydrogenase (*IDH1/IDH2*) mutation and 1p/19q codeletion were proposed to resolve oligoastrocytoma as either oligodendroglioma or astrocytoma^6^. In addition, *IDH1/IDH2* mutation, 1p/19q codeletion, *TP53* mutation and *MGMT* promoter methylation were used for prognostic modeling and stratification into molecularly determined treatment groups^5^,^7^,^8^,^9^,^10^. However, some questions remain ambiguous. For instance, within WHO grade II diffuse astrocytomas (DA) the prognostic relevance of the molecular markers has remained debate^10^,^11^,^12^,^13^,^14^,^15^,^16^,^17^,^18^ (Supplementary Tables S1 and S2). Therefore, more reliable molecular markers for predicting the course of disease and outcome of gliomas are still needed.

DNA methylation at the 5-carbon position of cytosine (5mC) is the most extensively studied epigenetic modification in human cancer^19^. In 2009, breakthrough studies indicated that 5mC can be converted to 5-hydroxymethylcytosine (5hmC) by the ten eleven translocated (TET) enzymes^20^,^21^. HPLC-MS analysis and immunohistochemistry revealed that 5hmC is present with highest level in central nervous system^22^. Subsequent studies indicated that 5hmC is not merely serving as an intermediate of DNA demethylation, but also acts as a stable epigenetic marker^23^. Meanwhile, abundant evidence detected that 5hmC globally decreased in most human malignancies, including gliomas^24^,^25^,^26^,^27^,^28^,^29^,^30^,^31^. Initially, 5hmC loss in gliomas was proposed to be related with *IDH1/IDH2* mutations^26^. However, subsequently numerous trials from larger clinical samples argued against this claim^25^,^29^,^30^,^31^. It was interesting that 5hmC loss were suggested to be prognostic for malignant gliomas (World Health Organization grade III or IV)^29^. Due to small sample and lack detailed information about management and adjuvant treatment in this study^29^, much more work needs to verify the prognostic value of 5hmC in gliomas. Here, we performed immunohistochemical investigation in 287 glioma cases with a well identified homemade anti-5hmC antibody. The results showed that 5hmC was an prognostic marker confined to DA but not grade III or IV glioma patients. Moreover, we detected that *IDH1* mutation by DNA sequencing and the combination of 5hmC/KI67 was associated with prognosis of DA respectively.

## Results

### Patient characteristics

The clinicopathological characteristics of the patients were summarized in Table 1. In total 287 patients, 143 (50%) cases were no more than age 40 with median age 41 (ranged from 16–76). The patient group consisted of 166 (58%) males and 121 (42%) females. Most gliomas (89%) located in the supratentorial areas. There were 23 (8%) grade I, 130 (45%) grade II, and 69 (24%) grade III and 64 (23%) grade IV glioma cases respectively. In the subtypes, most cases (33%) were DA. The overall follow-up durations ranged from 2 to 103 months (median, 24 months). A total of 144 (50%) patients were alive at the end of the follow-up, while 143 (50%) patients died of gliomas. The preoperative KPS scores of 179 (62%) patients were more than 80. Tumor volumes of 130 (45%) cases were less than 50 cm^3^. 212 (74%) cases had total tumor resection and 75 (26%) cases had subtotal tumor resection. Subsequent to surgery, 118 (41%) patients received combined radiotherapy and chemotherapy. 26 (9%) and 68 (24%) patients were treated with either radiotherapy or chemotherapy respectively. 75 (26%) patients did not receive further therapy.

### Identification of anti-5hmC antibody

**Figure 1:**
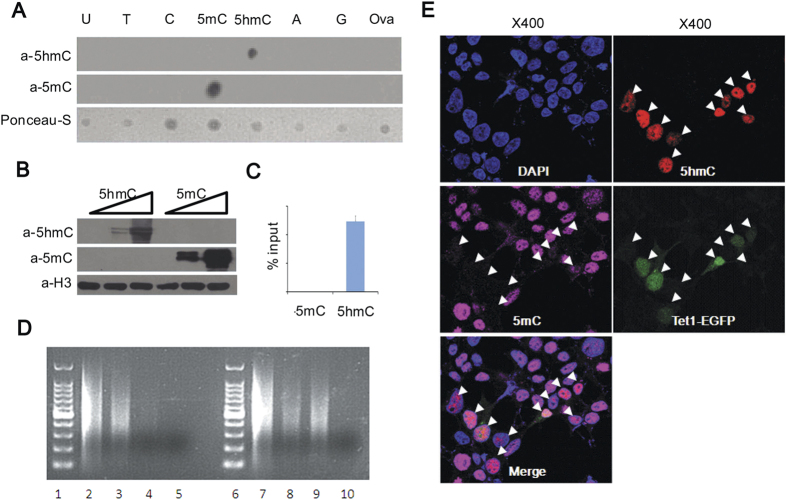
Identification of the specificity of anti-5hmC antibody. (**A**) Dotblot analysis. Equal conjugated bases were loaded and immunnoblotted with an antibody against 5hmC (top) or 5mC (middle). Loading was shown by Ponceau staining (bottom). (**B**) Western blotting. 0, 5, 50 pg of nucleoside-conjugated-ovalbumin was mixed with 293T cell lysate and separated by SDS-PAGE and immunnoblotted with anti-5hmC (upper), anti-5mC (middle) and anti-H3 antibodies (bottom). (**C**) qPCR quantifying immunoprecipitated DNA fragments containing 5mC or 5hmC by the anti-5mC (left) or anti-5hmC (right) antibody. (**D**) Denatured fragmented DNA from Tet1 nontransfected (Lane 2–5) or transfected (Lane 7–10) 293T cells was immunoprecipitated by an antibody for 5mC (lanes 3 and 8), 5hmC (Lanes 4 and 9), or control IgG (Lanes 5 and 10). Lanes 1 and 6 are 100-bp DNA marker and lanes 2 and 7 have 10% of DNA input. (**E**) Immunofluorescence analysis. 293T cells transfected with cDNA encoding the Tet1 catalytic domain-IRES-GFP (middle right, arrowheads) were stained with DAPI (top left), anti-5hmC antibody (top right), or anti-5mC antibody (middle left). Merged Image was in bottom left.

To evaluate specificity of anti-5hmC antibody generated by our lab, we firstly performed dot-blot analysis. The result showed that the rabbit polyclonal anti-5hmC antibody specifically recognized 5hmC instead of other bases (Fig. 1A). West-blot and immunoprecipitation also confirmed this result (Fig. 1B–D). Immunofluorescence analysis showed strong 5hmC staining was in the nucleus of *Tet1* transfected cells. Concomitantly, the level of 5mC decreased in *Tet1* transfected cells (Fig. 1E). Therefore, these results strongly demonstrated the anti-5hmC antibody had high specificity to recognize 5hmC.

### Relations between 5hmC reduction and clinicopathological features

**Figure 2:**
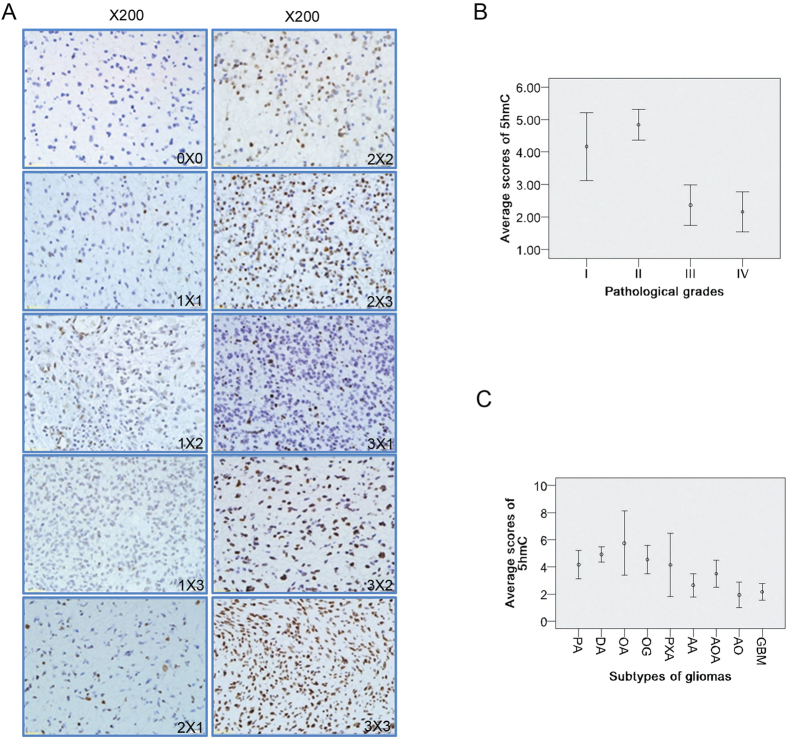
5hmC level examined by immunohistochemistry, and its relations with pathological grades or subtypes of gliomas. (**A**) Representative images of 5hmC immunostaining in glioma tissues. 200×. (**B,C**) Relations between 5hmC level and pathological grades (**B**) or subtypes (**C**) of gliomas. Error bar represents the standard error (SE).

5hmC level was analyzed by immunohistochemistry (IHC) in 287 glioma cases. In glioma tissues some samples had strong staining while other had weak or no staining, and cells in the same sample also had different degrees of staining intensity (Fig. 2A). To facilitate the analysis of immunohistochemical results, specific nuclear immunoreactivity was scored using a 9-point scale on the basis of the product of staining intensity (no staining = 0, weak staining = 1, moderate staining = 2, strong staining = 3), and staining extent (% of positive cells; <5% = 0, 5%–30% = 1, 30%–60% = 2, >60% = 3)^32^. To facilitate statistical analysis, we divided the samples into 2 groups according to staining scores. Group 1 had no or weak staining with the scores of 0 to 3. Group 2 had moderate and strong staining with the scores from 4 to 9. The relations between 5hmC level and clinicopathological features were summarized in Table 2. On Chi square analysis, the 5hmC reduction (scores 0–3) were significantly associated with the following variables: over age 40 (*P* < 0.001), high pathological grades (grade III and IV, *P* < 0.001), vital status (death, *P* < 0.001), lower KPS scores (<80, *P* = 0.018) and larger tumor size (≥50 cm^3^, *P* = 0.015). Nonsignificant variables included gender (*P* = 0.422), extent of resection (*P* = 0.225) and adjuvant treatment (*P* = 0.544). The average scores in grade I, II, III, and IV for 5hmC were 4.167 ± 0.524, 4.839 ± 0.235, 2.362 ± 0.310 and 2.156 ± 0.310 respectively (Fig. 2B). Spearman correlation analysis showed there was a significantly inverse correlation between the pathological grades of gliomas and the 5hmC scores (*r* = −0.407, *P* < 0.001).

For subtypes of gliomas, the average scores in pilocytic astrocytomas (PA); diffuse astrocytomas (DA); oligoastrocytomas (OA); oligodendrogliomas (OG); pleomorphic xanthoastrocytomas (PXA); anaplastic astrocytomas (AA); anaplastic oligoastrocytomas (AOA); anaplastic oligodendrogliomas (AO) and glioblastoma multiforme (GBM) for 5hmC were 4.167 ± 0.524, 4.926 ± 0.278, 5.750 ± 1.181, 4.542 ± 0.521, 4.143 ± 1.164, 2.649 ± 0.427, 3.500 ± 0.500, 1.933 ± 0.474 and 2.156 ± 0.310 respectively (Fig. 2C). Kruskal-Wallis test revealed significant difference between these groups (X^2^ = 61.678, *P* < 0.001).

### 5hmC level on patient survival in total glioma cases

**Figure 3:**
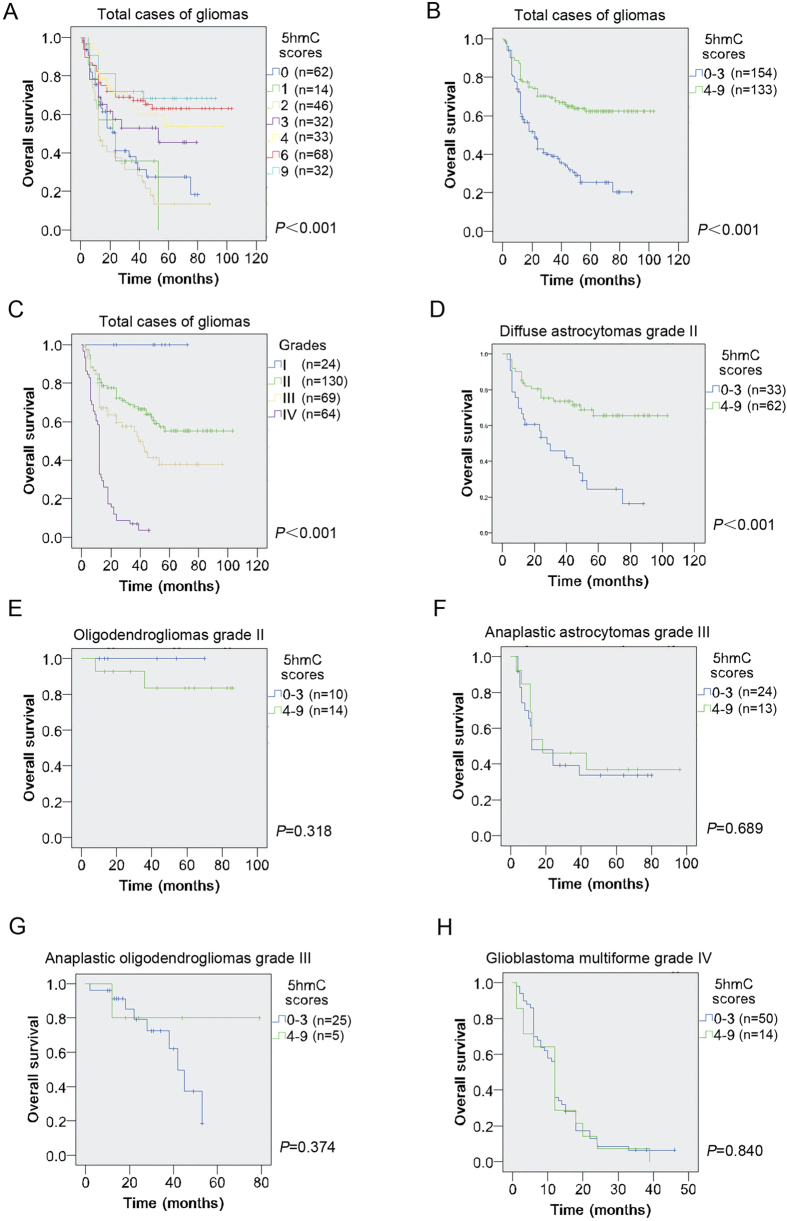
Prognostic relevance between 5hmC level and glioma patients by Kaplan-Meier analysis. (**A**,**B**) Kaplan-Meier survival analysis of glioma patients when all samples were divided into 7 groups (**A**) based on 5hmC scores 0, 1, 2, 3, 4, 6 and 9, or two groups (**B**) based on 5hmC scores 0–2 and 3–9. (**C**) Prognostic relevance between pathological grades and glioma patients was examined by Kaplan-Meier analysis. (**D–H**) Relevance between 5hmC level and patient survival within diffuse astrocytomas (**D**); oligodendrogliomas (**E**); anaplastic astrocytomas (**F**); anaplastic oligodendrogliomas (**G**) and glioblastoma multiforme (**H**) was tested by Kaplan-Meier analysis.

To determine the relations between 5hmC level and the survival of the glioma patients, we firstly divided the samples into seven groups according to the 5hmC score 0, 1, 2, 3, 4, 6 and 9. Kaplan-Meier survival analysis revealed low 5hmC scores strongly correlated with poor prognosis while high 5hmC scores correlated with better survival (Fig. 3A; χ^2^ = 40.570, *P* < 0.001). Next, we divided glioma patients into two groups. The cases with scores of 0 to 3 were defined as group 1, while patients with scores from 4 to 9 group 2. On Kaplan-Meier survival analysis, 5hmC reduction group statistically significantly correlated with poorer survival of patients, while 5hmC positive group with the better prognosis (Fig. 3B, χ^2^ = 31.109, *P* < 0.001). In addition, as previous reports^1^,^2^, higher pathological grade correlated with worse prognosis in total cases (Fig. 3C, χ^2^ = 124.243, *P* < 0.001).

To further evaluate the prognostic quality of 5hmC level, univariate and multivariate Cox regression analyses were performed. Univariate analysis (Table 3) confirmed the association of low 5hmC level with shorter patient survival time (*P* < 0.001). Additionally, the following variables were also significantly associated with poorer overall survival: aged over 40 at diagnosis (*P* < 0.001), higher pathological grades (*P* < 0.001), worse KPS (*P* = 0.026), larger tumor size (*P* = 0.014), lack adjuvant treatment after surgery (*P* = 0.032) and subtotal resection (*P* = 0.049). Nonsignificant variables included gender (*P* = 0.068) and tumor locations (*P* = 0.155). However, when the data were analyzed in the multivariate Cox regression model (Table 3), low 5hmC level was not found to have independent prognostic power (*P* = 0.720), while higher pathological grades (*P* < 0.001), lack adjuvant treatment (*P* = 0.009) and extent of resection (*P* = 0.017) remained statistically significant. In an alternative multivariate model exclusive of pathological grades, a strong interaction was found between 5hmC level and pathological grades (Table 3).

### 5hmC level on patient survival in subtypes of gliomas

Given the significant interaction between pathological grades and 5hmC level, we next analyzed the relations between 5hmC level and the patient survival within subtypes of gliomas. Kaplan-Meier survival analysis revealed low 5hmC scores were strongly associated with unfavorable survival within DA (Fig. 3D, χ^2^ = 14.788, *P* < 0.001) but not OG (Fig. 3E, χ^2^ = 0.997, *P* = 0.318), AA (Fig. 3F, χ^2^ = 0.160, *P* = 0.689), AO (Fig. 3G, χ^2^ = 0.790, *P* = 0.374) and GBM (Fig. 3H, χ^2^ = 0.041, *P* = 0.840). The median survival time for DA with low 5hmC level was 37.66 (95% CI, 26.66 ∼ 48.67) months, while that with moderate and high 5hmC level was 75.25 (95% CI, 64.90 ∼ 85.62) months.

**Figure 4:**
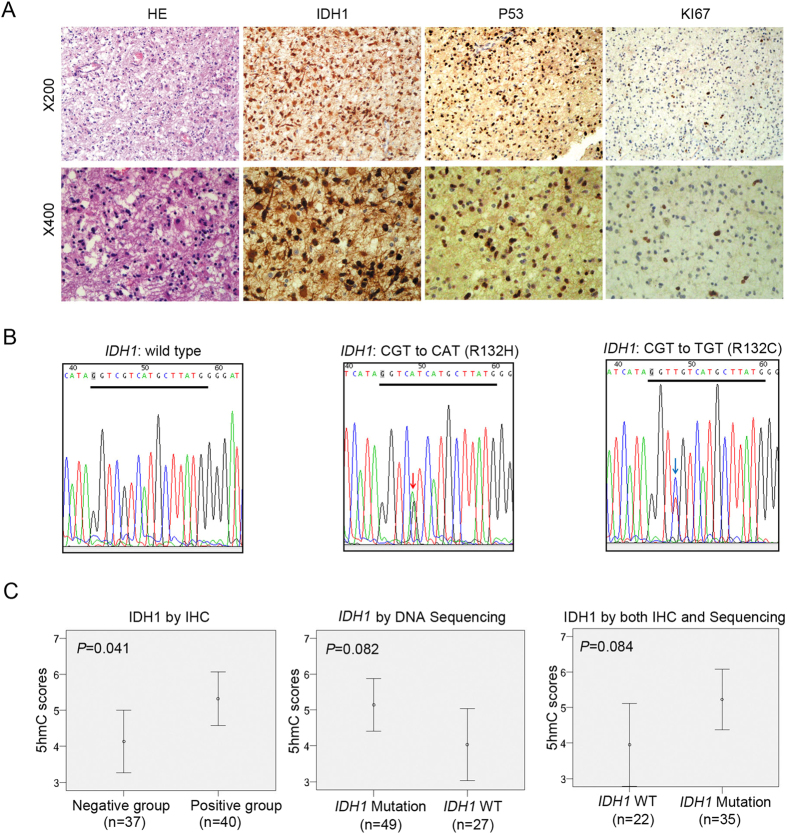
IDH1 mutation status is not associated with 5hmC reduction. (**A**) HE and representative immunostaining images of IDH1, P53 and KI67 in diffuse astrocytomas. 200× (upper), 400× (bottom). (**B**) Representative *IDH1* wild-type (WT), *IDH1* (red arrow, R132H) and *IDH1* (blue arrow, R132C) mutations were analyzed by Sanger sequencing. (**C**) Comparison of 5hmC level in DA cases between with *IDH1* mutation and wild type by immunohistochemistry (IHC), DNA sequencing, or both IHC and DNA sequencing using the unpaired Student’s t-test. Error bar represents the standard error.

To compare the prognostic value of 5hmC with other well-known prognostic markers within DA, we examined the level of IDH1, p53 and KI67 by immunohistochemistry in these tumors (Fig. 4A). Available data for IDH1, P53 and KI67 were 77, 69 and 71 cases respectively. 52% (40 of 77) of DA cases showed IDH1-R132H positive immunostaining. Immunodetection of P53-positive or KI67-positive cell nuclei ranged up to 80% or 30% respectively. According to the 10% cutoff, P53-positive cases were detected in 46% (32 of 69) of DA patients. With cutoff as 4%, KI67-positive cases were 35% (25 of 71) of DA cases. We also examined the mutation status of *IDH1/2* by direct DNA sequencing and detected 64% (49/76) of DA bear heterozygous *IDH1* mutations (Fig. 4B). The predominant amino acid sequence alteration in IDH1 was R132H accounting for 98% (48/49) of the detected mutations. Only one case was found to bear IDH1-R132C mutation (Fig. 4B). We did not detect codon 172 of IDH2 mutation at present study (data not shown). Of 70 samples by both DNA sequencing and immunohistochemistry assays, 45 cases were identified as carrying *IDH1* mutation by DNA sequencing. However, only 78% (35/45) DA with *IDH1* mutation by DNA sequencing was positive for IDH1-R132H antibody. The three cases with positive immunoreactivity for IDH1-R132H antibody did not show *IDH1/2* mutation by DNA sequencing.

The relations 5hmC level on IDH1 immunostaining signaling, IDH1 mutated status, P53 and KI67 label index and patient clinicopathological features were outlined in Table 4. Chi square analyses confirmed significant association between 5hmC level and vital status (*P* < 0.001). Additionally, although the significant correlation between 5hmC level and IDH1 mutation was reached (*P* = 0.036), most cases with low 5hmC scores were detected in the IDH1 wild type cases (61%, 30 of 49). We investigated the relationship between *IDH1* mutation status and 5hmC levels. Surprisingly, we detected that 5hmC reduction seem to be associated with *IDH1* wild type versus mutation cases (Fig. 4C). Thus, this result was consistent with previous conclusions that 5hmC reduction didn’t result from *IDH1* mutation^25^,^29^,^30^.

To further test the 5hmC prognostic value for DA patients, we performed univariate and multivariate analyses using a Cox proportional hazards model. Consistent with Kaplan-Meier and Chi square analysis, univariate and multivariate analysis verified low 5hmC level was associated with unfavorable survival of DA patients (Table 5, *P* < 0.001,*P* = 0.013). In addition, univariate Cox regression analyses also revealed that DA patients with either positive immunoreactivity for IDH1-R132H antibody (*P* = 0.030) or *IDH1* mutation by DNA sequencing (*P* = 0.001) had a better survival (Table 5). However, multivariate analyses revealed that *IDH1* mutation by DNA sequencing (*P* = 0.021) versus IDH1 positive immunstaining (*P* = 0.254) had an prognostic value for DA patients (Table 5). In addition, we found that neither P53 nor KI67 harbored prognostic power by uni- or multivariate analysis (Table 5).

### Molecular markers in combination for prognostic relevance within DA

**Figure 5:**
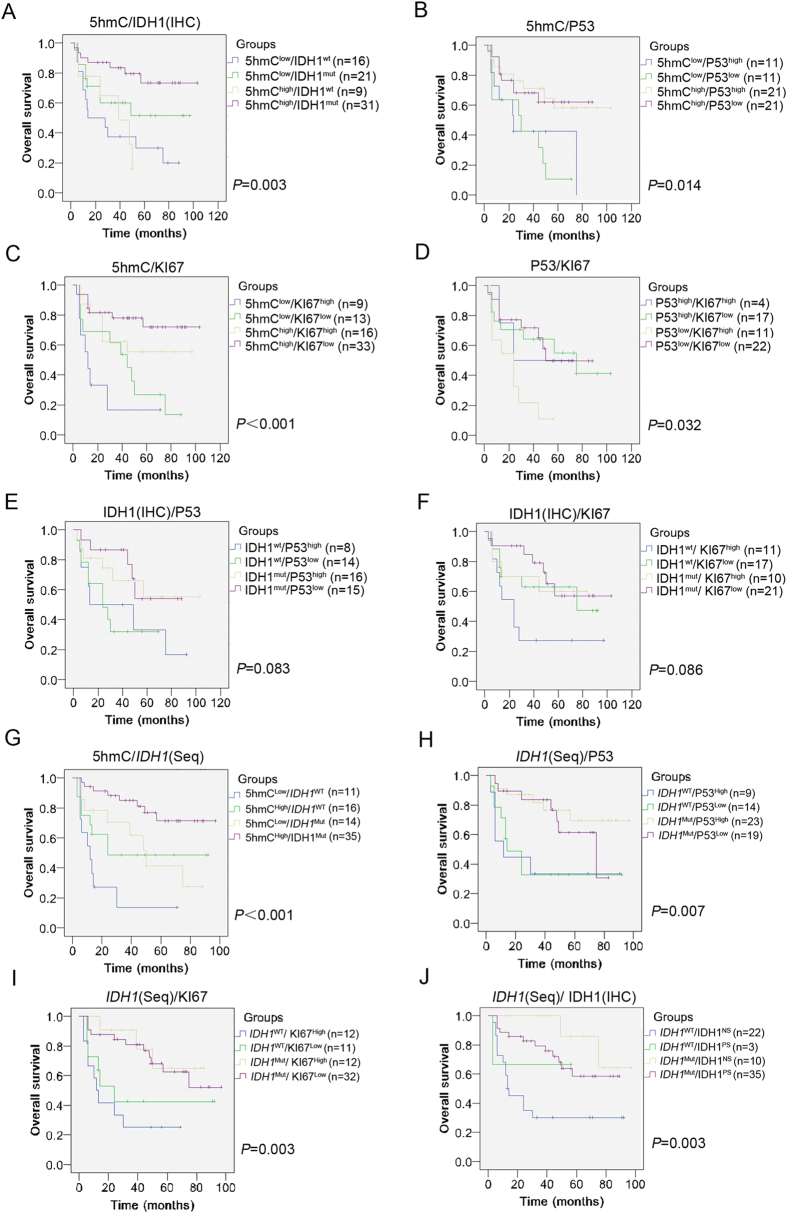
Overall survival by molecular markers in combination within diffuse astrocytomas. (**A–J**) Relevance between patient survival and subgroups of 5hmC/IDH1 (IHC) (**A**), 5hmC/P53 (**B**), 5hmC/KI67 (**C**), P53/KI67 (**D**), IDH1 (IHC)/P53 (**E**), IDH1 (IHC)/KI67 (**F**), 5hmC/*IDH1* (Seq) (**G**), *IDH1* (Seq)/P53 (**H**), *IDH1* (Seq)/KI67 (**I**) and *IDH1* (Seq)/IDH1 (IHC) (**J**) was tested by Kaplan-Meier analysis. IHC: immunohistochemistry. Seq: sequencing.

To test whether combination of 5hmC, IDH1, P53 or KI67 can be applied for assessment of prognosis and restratification of DA, we stochastically divided patients into subgroups with two molecular combinations. Kaplan-Meier survival analysis revealed combinations of 5hmC/IDH1 (IHC)(Fig. 5A, X^2^ = 13.661, *P* = 0.003), 5hmC/P53 (Fig. 5B, X^2^ = 10.616, *P* = 0.014), 5hmC/KI67 (Fig. 5C, X^2^ = 17.717, *P* = 0.000), P53/KI67 (Fig. 5D, X^2^ = 8.776, *P* = 0.032), 5hmC/*IDH1* (Seq) (Fig. 5G, X^2^ = 14.312, *P* = 0.000), *IDH1* (Seq)/P53 (Fig. 5H, X^2^ = 11.113, *P* = 0.007), *IDH1* (Seq)/KI67 (Fig. 5I, X^2^ = 12.989, *P* = 0.003) and IDH1 (IHC)*IDH1* (Seq) (Fig. 5J, X^2^ = 11.412, *P* = 0.003) were associated with OS. Nonsignificant combinations included IDH1 (IHC)/P53 (Fig. 5E, X^2^ = 6.667, *P* = 0.083) and IDH1 (IHC)/KI67 (Fig. 5F, X^2^ = 6.582, *P* = 0.086). Surprisingly, the group for best assessment of prognosis and restratification of DA was 5hmC/KI67 combinations (Table 6). Within this group, the mean OS for 5hmC^low^/KI67^high^, 5hmC^low^/KI67^low^, 5hmC^high^/KI67^high^ and 5hmC^high^/KI67^low^ groups were 22 (95% CI, 6–38), 42 (95% CI, 26–58), 63 (95% CI, 44–82) and 80 (95% CI, 67–94) months respectively. In contrast, the mean OS for 5hmC^low^/IDH1^NS^, 5hmC^low^/IDH1^PS^, 5hmC^high^/IDH1^NS^ and 5hmC^high^/IDH1^PS^ groups were 37 (95% CI, 21–53), 59 (95% CI, 41–77), 35 (95% CI, 22–48) and 83 (95% CI, 69–96) months respectively. Even for 5hmC/*IDH1* (Seq) combinations, the mean OS for 5hmC^Low^/*IDH1*^WT^, 5hmC^Low^/*IDH1*^Mut^, 5hmC^High^/*IDH1*^WT^ and 5hmC^High^/*IDH1*^Mut^ groups were 20 (95% CI, 6–33), 51 (95% CI, 34–68), 51 (95% CI, 30–70) and 79 (95% CI, 67–89) months respectively. Therefore, the combination of 5hmC/KI67 was a useful prognostic marker for restratification of DA.

## Discussion

The majority of the DA exhibits a relatively good prognosis. However, some DA show unexpectedly aggressive clinical course leading to early patient death^10^. This creates a diagnostic dilemma for routine histopathology. The heterogeneous clinical courses of DA may be associated with different epigenetic and genetic abnormalities^33^. Therefore, molecular markers would be useful for the accurate restratification of these tumors and provide help for prognostication or therapeutic decision making. However, currently, available molecular markers are limited.

Epigenetic modifications play crucial roles in normal development and frequently alter during carcinogenesis^33^. 5hmC was a new detected DNA modification and the knowledge about its roles in gliomas remained limited. With an identified homemade anti-5hmC antibody, we detected that 5hmC variably reduced in most gliomas and the reduction was closely associated with higher pathological grades. These findings were consistent with previous reports^24^,^25^,^28^,^29^,^30^. In clinical practice, it still remains a challenge for pathologists to histologically define AA and DA based on morphological features of anaplasia, such as mitotic activity and microvascular proliferation, for poor interobserver agreement^34^. *IDH1/2* mutation has been accepted as a favorable prognostic biomarker for gliomas^5^. However, *IDH1/2* mutated status can’t be used to differentiate AA from DA because both tumors have high rates of *IDH1/2* mutation. In the present study, we detected 5hmC level decreased more severely in grades III/IV versus I/II gliomas (Fig. 2B). This means 5hmC loss may be associated with anaplastic progression and can be used to stratify low-grade gliomas (I/II) from anaplastic gliomas (III/IV). In addition, we also detected that 5hmC dramatically reduced in AA and GBM compared to DA (Fig. 2C). Therefore, 5hmC loss may be a usefully marker for differentiating AA from DA.

Apart from the finding that 5hmC loss was a marker for anaplastic progression of gliomas, we also detected that 5hmC reduction was closely associated with shortened survival of glioma patients. However, in multivariate analysis, 5hmC had no independent prognostic value in the entire patient cohort. Alterative multivariate model found that pathological grade was the major interacting factor (Table 3). Further analysis within subtypes of gliomas revealed that 5hmC was still a prognostic marker confined to diffuse astrocytomas WHO II (Fig. 3D, Table 5). Inconsistent with Orr *et al.* report^29^, we didn’t find prognostic relevance between 5hmC level and AA or GBM (Fig. 3F, H). The discrepancy may be associated with sample size (only 12 cases of AA with prognostic data in Orr *et al.* study), evaluation of 5hmC level methods, patient races or choice of treatment. Although Orr *et al.* had 52 adult GBM for evaluating prognostic relevance with 5hmC level, the short survival time (mean 15 months) and most cases without detected 5hmC may limited the 5hmC prognostic value within GBM. In addition, only 10 cases with prognostic data in Orr’s study may account for their failed detection of the relevance between 5hmC level and DA.

Many studies have confirmed an association of *IDH1* mutations with favorable outcome for patients with malignant gliomas (WHO III/IV)^7^,^35^,^36^,^37^. However, prognostic value of *IDH1* mutations for low-grade gliomas (LGG, WHO II), especial DA, was subject to debate^11^,^14^,^17^,^38^,^39^ (supplementary Table S1 and S2). For example, in a largest series IDH1 was of no prognostic value for 360 patients suffering from LGG, in which 186 was oligodendroglial tumors and 174 astrocytic tumors^17^. A recent Cancer Genome Atlas Research showed *IDH* mutations had significant survival prediction in lower grade gliomas (WHO II and III), including oligodendroglioma, oligoastrocytoma and astrocytoma. However, the report didn’t particularly analyze prognostic value of *IDH1* mutations within DA (WHO II)^38^. Ahmadi *et al.* performed *IDH1/2* mutation assays in a series of 100 DAs patients and detected no survival benefit of *IDH1* mutations in these patients^11^. However, by immunohistochemistry, two groups recently independently found positive IDH1 staining still hold prognostic relevance with DA patients^14^,^39^. In the present study, both univariate and multivariate Cox regression analyses confirmed that *IDH1* mutation by DNA sequencing was associated with better survival for DA patients (Table 5). Therefore, our results suggested that *IDH1* mutation was still a favorable prognostic marker for DA.

Currently, IDH1, TP53 and KI67 are routinely used molecular markers for assisting prognostic decision^5^,^10^,^40^,^41^,^42^ (supplementary Table S1). To test the prognostic value of combination of 5hmC, IDH1, P53 or KI67, we stochastically divided patients into subgrokups with two molecular combinations in DA. It is interesting that the combination of 5hmC/KI67 harbored the best value for assessment of prognosis and restratification of DA. (Fig. 5, Table 6). The reasons for 5hmC/KI67 but not other combinations in best predicting DA patient survival are unknown. One possible reason is that 5hmC/KI67 is a combination of cell differentiation (5hmC) and proliferation (KI67) markers. Several lines demonstrated that differentiated cells instead of stem cells harbor high 5hmC level^24^,^29^. In addition, an inverse relationship between 5hmC levels and cell proliferation was detected in proliferating or differentiated cells^25^. Functionally, double-knockout of *Tet1* and *Tet2* resulted in reduced 5hmC level and delayed brain development^43^. Therefore, the complementary combination of 5hmC and KI67 may be reason for their best assessment of prognosis and restratification of DA. It is interesting that the emerging data for superior prognosis of ATRX/IDH co-mutant diffuse astrocytomas was recently addressed in some reports^38^. Further work needs to compare prognostic value between 5hmC/KI67 and ATRX/IDH within DA.

Previously, some reports suggested *IDH1* mutations might account for 5hmC reduction in gliomas by means of the presumed role of 2-hydroxyglutarate as an inhibitor of TET oxidases^26^. However, this suggestion was challenged by other observations that 5hmC reduction not associated with IDH1 mutations^25^,^29^,^30^. In the present study, we detected a higher versus lower level of 5hmC in *IDH1* mutated DA compared to *IDH1* wild type tumors (Fig. 4C). Therefore, our results didn’t support 5hmC reduction was associated with *IDH1* mutations. This conclusion was further supported by the observation that 5hmC loss was associated with poorer prognosis (Fig. 3A,B,D), while *IDH1* mutations correlated with better survival in DA (Table 5). Since 5hmC can be converted from 5mC by TET enzymes^20^,^21^, 5hmC loss may be associated with 5mC reduction in malignancies. However, Kraus *et al.* observed that 5hmC level was unrelated to 5mC values by isotope-based liquid chromatography mass spectrometry assays^28^. Therefore, 5hmC decrease in cancer cells may primarily result from the alterations of *TET* genes. Indeed, one of *TET* family genes, *TET2*, was detected with high mutation rates in some hematologic malignancies, which simultaneously had aberrant levels of 5hmC in their genomes^44^,^45^. Some other evidence showed that 5hmC loss was associated with decreased expression or nuclear exclusion of TETs proteins^29^,^30^. Much more work need to confirm the relations between TETs alteration and 5hmC reduction in gliomas.

Summarizing, our data suggested that the 5hmC level, *IDH1* mutation and 5hmC/KI67 combination harbor the value for assessment of prognosis of DA. Some limitations existed in this study. These data are derived from an unselected single-center collective. The sample size for each entity was not large, the design was in retrospective, and choice of treatment was not standardized. Thus, the prognostic value of 5hmC level, *IDH1* mutation and 5hmC/KI67 combination in DA needs further verify.

## Methods

### Sample collection and clinical follow-up

Samples collection and analysis were approved by the ethics committee of the Xijing Hospital, Fourth Military Medical University, Xi’an, P. R. China. Written informed consent was obtained from all of the patients. All specimens were handled and made anonymous according to the ethical and legal standards. To collect samples, patients who died of diseases not directly related to gliomas were excluded. After that, a total of 287 glioma samples with 2–10 years follow-up information were collected from Xijing Hospital, Fourth Military Medical University between 2003 and 2013. Patients’ clinicopathological features such as age at diagnosis, gender, tumor size, Karnofsky performance status (KPS) score, extent of resection and adjuvant treatment were collected. Three pathologists independently reviewed all histological slides and graded each glioma according to 2007 World Health Organization criteria. In case of a discrepancy, the 3 observers simultaneously reviewed the slides to achieve a consensus. All methods were carried out in accordance with the approved guidelines of Fourth Military Medical University.

### Production and examination of anti-5hmC antibody

To examine 5hmC level in glioma tissues, we produced polyclonal rabbit anti-5hmC antibody. The specificity of the anti-5hmC antibody was examined by dot blot, western blot, immunoprecipitation and immunofluorescence. In brief, each nucleoside was conjugated to ovalbumin and quantified by mass spectrometrical analysis. Equal conjugated bases were spotted onto nitrocellulose filter membrane and reacted with the 5hmC (1:1000) or 5mC (1:500, Calbiochem) antibody. For western blot, 5mC or 5hmC conjugated with ovalbumin was mixed with 293T cell lysates and separated by SDS-PAGE and immunoblotted. For immunoprecipitation assay, a 367-bp DNA fragment spanning 4605333-4605699 in mouse chromosome 10 was PCR-amplified with 5mC or 5hmC in place of cytodine. 8.7 ng PCR products was mixed with 5μg sonicated genomic DNA of 293T cells. Then 2.5 μg of 5hmC antibody was added to the denatured DNA for precipitation. IP-DNA was extracted for quantitative reverse transcriptase PCR or regular PCR. To test whether the antibody could be used for immunofluorescence analysis, we transfected cDNA encoding the Tet1 catalytic domain into 293T cells by mean of that Tet1 can convert 5mC to 5hmC. Subsequently, immunofluorescence was performed with fluorescence-conjugated secondary antibody (1:500; Santa Cruz) as described.

### Immunohistochemistry (IHC) analysis

Immunohistochemistry for 5hmC was performed in tissue sections of glioma samples as described previously^32^. In brief, Paraffin embedded tissue blocks were cut to 4μm sections and deparaffinized and rehydrated using xylene and ethanol; 3% H_2_O_2_ in phosphate buffered saline (PBS) was used to inactivate the endogenous peroxidase. DNA was denatured by immersing sections in 2 N HCl for 30 minutes then neutralizing in boric acid buffer for 10 minutes at room temperature. The slides were blocked with goat serum to reduce nonspecific binding and then incubated with primary 5hmC (1:1000 dilution) antibody overnight at 4 °C. On the following day, the peroxidase-conjugated secondary antibody (1:500 dilution; Santa Cruz) was incubated for 1 hour at room temperature. Diaminobenzidine (DAB) substrate was used for detection and hematoxylin was used for counterstaining. The samples were then mounted for visualization. The cells with brown nuclei were considered positively stained. The level of 5hmC staining was accessed independently by 3 pathologists.

Immunohistochemistries for IDH1-R132H (H09, Dianova, Hamburg, Germany; dilution 1:100), P53 (DO-7, Dako, Carpinteria,CA, USA; dilution 1:100) and KI67 (MIB-1, Dako, Glostrup, Denmark; dilution 1:50) were performed as described above exclusive the step of DNA denature by HCl. Each slide stained for IDH1-R132H, P53 and KI-67 was individually reviewed and scored by 3 independent observers. Microscopic areas with highest labeling intensity were chosen for calculation. The p53 or KI-67 labeling index (LI) was defined as the percentage of immunoreactive tumor cell nuclei. In each case either at least 1000 tumor cell nuclei were counted were examined. For statistical analysis, cutoff value of LI for P53 and KI67 was 10% and 4% respectively according to previous reports^41^,^42^,^46^.

### PCR amplification and genes sequencing

For *IDH1* and *IDH2* mutations assays, DNA extraction from formalin fixed paraffin embedded tissue was used. Tumor content of at least 80% was histologically determined for each sample used for DNA extraction. Nucleic acid extraction was performed by standard procedures. 150 ng of genomic DNA was used for PCR amplification in a total volume of 50 μl. The primer sequences for *IDH1* and *IDH2* and PCR amplification conditions were described previously^47^. IDH1 codon 132 and IDH2 codon 172 were analyzed by direct sequencing. If results were ambiguous the *IDH1* or *IDH2* were amplified by use of a different set of primers as described previously^47^.

### Statistical Analysis

We used SPSS 20.0 (SPSS Inc. Chicago, IL, USA) for the statistical analysis. The associations between 5hmC level and clinicopathological features were compared by using a Chi square test or Fisher’s exact test. The nonparametric Spearman correlation was used to analyze the relationship between pathological grades of glioma and 5hmC levels. Overall survival (OS) was defined as the interval between the date of diagnosis and the date of death or the last known follow-up. OS data were censored at the date of last follow-up, if the patient was still alive. Kaplan-Meier survival analysis was used to present the relationship between patient survival and 5hmC levels. Survival differences were analyzed by the log-rank test. Univariate and Multivariate analyses were performed using a Cox proportional hazards model to identify independent prognostic factors. *P*-values were all 2-sided and used as significance threshold less than 0.05.

## Additional Information

**How to cite this article**: Zhang, F. *et al.* 5-hydroxymethylcytosine loss is associated with poor prognosis for patients with WHO grade II diffuse astrocytomas. *Sci. Rep.* **6**, 20882; doi: 10.1038/srep20882 (2016).

## Supplementary Material

Supplementary Information

## Figures and Tables

**Table 1:** Patient Characteristics.

Total cases	N = 287	%
Age in years		
Median. range	41 (16 ∼ 76)	
≤40:>40	143:144	50:50
Gender		
Male: Female	166:121	58:42
Glioma location		
Supratentorial: other*	254:33	89:11
WHO grades		
I: II: III: IV	24:130:69:64	8:45:24:23
Subtypes		
PA(GI)	24	8
DA:OA:OG:PXA(GII)	95:4:24:7	33:2:8:2
AA:AOA:AO(GIII)	37:2:30	14:1:10
GBM(G IV)	64	22
Vital status		
Alive:death	144:143	50:50
≥80:<80	179:108	62:38
^1^Tumor size (cm)		
<50 cm^3^: ≥ 50cm^3^: lack data	130:130:27	45:45:10
Extent of resection		
Total:subtotal	212:75	74:26
Adjuvant treatment		
RC:R:C:L	118:26:68:75	41:9:24:26

^*^other: cerebellum, pons, brain stem and spinal cord.

PA, pilocytic astrocytoma; DA, diffuse astrocytoma; OA, oligoastrocytoma; OG, oligodendroglioma; PXA, pleomorphic xanthoastrocytoma; AA, anaplastic astrocytoma; AOA, anaplastic oligoastrocytoma; AO, anaplastic oligodendroglioma; GBM, glioblastoma multiforme. GI, grade I; GII, grade II; GIII, grade III; G IV, grade IV.

RC, radiation and chemotherapy; R, radiation therapy; C, chemotherapy; L, lack adjuvant treatment.

^1^Available data were 260 cases.

**Table 2:** Relationship between the 5hmC level and clinicopathological features of patients with gliomas.

Clinicopathological features	Total cases (n = 287)		
	5hmC (0–3)	5hmC (4–9)	*P*value
Gender			
Male: Female	93:62	73:59	0.422
Age			
≤40:>40	61:94	82:50	0.000
WHO grade			
I∼II: III∼IV	54:101	100:32	0.000
Vital status			
Alive:death	59:96	85:47	0.000
KPS			
≥80:<80	87:68	92:40	0.018
*Tumor size (cm)			
<50 cm^3^: ≥ 50cm^3^	60:95	70:62	0.015
Extent of resection			
Total:Subtotal	119:36	93:39	0.225
Adjuvant treatment			
Yes:No	113:42	99:33	0.544

^*^Available data were 260 cases.

**Table 3:** Univariate and multivariate analysis of variables associated with survival in total glioma cases (n = 287).

	Univariate analysis			Multivariate analysis			Alternative multivariate analysis		
	HR	95% CI	P-value	HR	95% CI	P-value	HR	95% CI	*P*-value
Gender									
(male vs female)	1.373	0.977–1.930	0.068	1.484	0.994–2.215	0.053	1.678	1.115–2.526	0.013
Age									
>40 vs ≤40)	1.956	1.396–2.742	<0.001	1.035	0.675–1.588	0.875	1.704	1.152–2.519	0.008
Location									
(supratentorial: other)	1.536	0.851–2.775	0.155	1.615	0.778–3.350	0.198	1.169	0.569–2.401	0.671
WHO grade									
(IV vs III. vs II vs I)	2.641	2.141–3.192	<0.001	2.641	2.064–3.380	<0.001			
KPS score									
(≤80 vs >80)	1.459	1.046–2.036	0.026	1.192	0.823–1.725	0.353	1.198	0.826–1.738	0.342
^1^Tumor size (cm)									
(>50cm^3^ vs ≤ 50cm^3^)	1.558	1.094–2.217	0.014	1.270	0.867–1.859	0.220	1.446	1.006–2.078	0.046
Extent of resection									
(subtotal vs total)	1.424	1.000–2.027	0.049	1.660	1.093–2.520	0.017	1.584	1.059–2.369	0.025
Adjuvant treatment									
(no vs yes)	1.472	1.034–2.095	0.032	1.703	1.142–2.539	0.009	1.731	1.139–2.630	0.010
5hmC scores									
(0–3 vs 4–9)	2.588	1.816–3.690	<0.001	1.081	0.706–1.654	0.720	1.837	1.235–2.733	0.003

HR, hazard ratio. ^1^Available data were 260 cases.

**Table 4:** Relationship between the 5hmC level and clinicopathological features of patients with diffuse astrocytomas (WHO grade II).

Molecular features	Total cases (n = 95)		
	5hmC (0–3)	5hmC (4–9)	*P*
Gender			
Male: Female	17:19	35:24	0.292
Age			
≤36: >36	16:20	30:29	0.673
Location			
Supratentorial: other	31:5	52:7	0.761
Vital status			
Alive:death	10:26	43:16	0.000
KPS			
≥80: <80	28:8	40:19	0.353
^1^Tumor size (cm)			
<50 cm^3^: ≥50 cm^3^	19:11	25:23	0.358
Extent of resection			
Subtotal :Total	13:23	20:39	0.828
Adjuvant treatment			
No:Yes	9:27	14:45	1.000
^2^IDH1 IHC			
Positive: Negative	10:18	30:19	0.036
^3^P53 label index			
≤10: >10	14:11	23:21	0.806
^4^KI67 label index			
≤4: >4	16:9	30:16	1.000
^5^IDH1 Sequencing			
Mutation: Wild type	14:11	35:16	0.315

^1–5^Available data were 78, 77, 69, 71 and 76 cases respectively.

IHC: immunohistochemistry.

**Table 5:** Univariate and multivariate analysis of variables associated with survival within diffuse astrocytomas (WHO grade II, n = 95).

	Univariate analysis			Multivariate analysis		
	HR	95% CI	*P*-value	HR	95% CI	*P*-value
Gender						
(male vs female)	1.449	0.790–2. 660	0.231	1.538	0.644–3.675	0.333
Age						
(> 40 vs ≤40)	1.103	0.601–2.028	0.751	1.358	0.555–3.323	0.503
Location						
(supratentorial: other)	1.155	0.486–2.784	0.744	1.189	0.345–4.094	0.784
KPS score						
(≤80 vs >80)	1.545	0.820–2.910	0.179	2.084	0.719–6.046	0.176
Adjuvant treatment						
(no vs yes)	1.369	0.701–2.675	0.358	1.425	0.576–3.525	0.444
Extent of resection						
(subtotal vs total)	1.232	0.640–2.370	0.533	1.225	0.475–3.164	0.674
^1^Tumor size (cm)						
(>50 cm^3^ vs ≤50 cm^3^)	1.490	0.781–2.843	0.226	1.709	0.549–5.325	0.355
5hmC score						
(0–3 vs 4–9)	3.822	2.038–7.168	0.000	3.343	1.296–8.622	0.013
^2^IDH1 IHC						
(Negative:Positive)	2.161	1.079–4.326	0.030	2.158	0.575–8.094	0.254
^3^P53 labeling index						
(High:Low)	1.175	0.590–2.342	0.646	1.040	0.407–2.660	0.934
^4^KI67 labeling index						
(High:Low)	1.532	0.778–3.017	0.217	1.527	0.522–2.458	0.439
^5^*IDH1* Sequencing						
(WT: Mutation)	3.066	1.537–6.116	0.001	5.630	1.304–24.302	0.021

^1–5^Available data were 78, 77, 69, 71 and 76 cases respectively. HR, hazard ratio; IHC: immunohistochemistry; WT: wild type.

**Table 6:** Molecular markers in combination for prognostic relevance within diffuse astrocytomas (WHO grade II).

Groups	Subgroups	Mean time (months) of OS (95% CI)	*P*-value
5hmC/IDH1 (IHC)	5hmC^Low^/IDH1^NS^: 5hmC^Low^/IDH1^PS^:	37 (95% CI, 21–53): 59 (95% CI, 41–77):	0.003
	5hmC^High^/IDH1^NS^: 5hmC^High^/IDH1^PS^	35 (95% CI, 22–48): 83 (95% CI, 69–96)	
5hmC/P53	5hmC^Low^/P53^High^: 5hmC^Low^/P53^Low^:	40 (95% CI, 19–60): 31 (95% CI, 17–44):	0.014
	5hmC^High^/P53^High^: 5hmC^High^/P53^Low^	71 (95% CI, 53–87): 62 (95% CI, 48–76)	
5hmC/KI67	5hmC^Low^/KI67^High^: 5hmC^Low^/KI67^Low^:	22 (95% CI, 6–38): 42 (95% CI, 26–58):	0.000
	5hmC^High^/KI67^High^: 5hmC^High^/KI67^Low^	63 (95% CI, 44–82): 80 (95% CI, 67–94)	
P53/KI67	P53^High^/KI67^High^: P53^High^/KI67^Low^:	45 (95% CI, 18–72): 62 (95% CI, 42–83):	0.032
	P53^Low^/KI67^High^: P53^Low^/KI67^Low^	22 (95% CI, 12–33): 60 (95% CI, 43–73)	
IDH1 (IHC)/P53	IDH1^NS^/P53^High^: IDH1^NS^/P53^Low^	41 (95% CI, 16–64): 33 (95% CI, 20–47):	0.083
	IDH1^PS^/P53^High^: IDH1^PS^/P53^Low^	68 (95% CI, 47–90): 64 (95% CI, 48–81)	
IDH1 (IHC)/KI67	IDH1^NS^/KI67^High^: IDH1^NS^/KI67^Low^	38 (95% CI, 16–60): 60 (95% CI, 42–79):	0.086
	IDH1^PS^/KI67^High^: IDH1^PS^/KI67^Low^	57 (95% CI, 36–78): 75 (95% CI, 58–91)	
5hmC/*IDH1* (Seq)	5hmC^Low^/*IDH1*^WT^: 5hmC^Low^/*IDH1*^Mut^:	20 (95% CI, 6–33): 51 (95% CI, 34–68):	0.000
	5hmC^High^/*IDH1*^WT^: 5hmC^High^/*IDH1*^Mut^	51 (95% CI, 30–70): 79 (95% CI, 67–89)	
*IDH1* (Seq)/P53	*IDH1*^WT^/P53^High^: *IDH1*^WT^/P53^Low^	37 (95% CI, 12–62): 38 (95% CI, 18–59):	0.007
	*IDH1*^Mut^/P53^High^: *IDH1*^Mut^/P53^Low^	76 (95% CI, 61–90): 61 (95% CI, 48–73)	
*IDH1* (Seq)/KI67	*IDH1*^WT^/KI67^High^: *IDH1*^WT^/KI67^Low^	26 (95% CI, 11–40): 45 (95% CI, 21–70):	0.003
	*IDH1*^Mut^/KI67^High^: *IDH1*^Mut^/KI67^Low^	68 (95% CI, 51–84): 70 (95% CI, 57–82)	
*IDH1* (Seq)/IDH1 (IHC)	*IDH1*^WT^/IDH1^NS^: *IDH1*^WT^/IDH1^PS^	36 (95% CI, 20–52): 38 (95% CI, 10–66):	0.003
	*IDH1*^Mut^/IDH1^NS^: *IDH1*^Mut^/IDH1^PS^	85 (95% CI, 71–99): 65 (95% CI, 54–76)	

OS, overall survival. IHC: immunohistochemistry; NS: negative staining; PS: positive staining; Mut: Mutation; WT: wild type; Seq: sequencing.
