# Supplementary material for: 5-hydroxymethylcytosine loss is associated with poor prognosis for patients with WHO grade II diffuse astrocytomas
Source: Sci Rep. 2016 Feb 11;6:20882. doi: 10.1038/srep20882 (PMC4749994; doi:10.1038/srep20882)
Supplement: Supplementary Information [file srep20882-s1.pdf]

5-hydroxymethylcytosine loss is associated with poor prognosis for patients with WHO grade II diffuse astrocytomas

Feng Zhang[1,*], Yifan Liu[2], Zhiwen Zhang[1], Jie Li[1], Yi Wan[3], Liying Zhang[1], Yangmei Wang[1], Xia Li[1], Yuqiao Xu[1], Xin Fu[1], Xiumin Zhang[1], Ming Zhang[4], Zhekai Zhang[4], Jing Zhang[1], Qingguo Yan[1], Jing Ye[1], Zhe Wang[1], Charlie Degui Chen[2], Wei Lin[5,*], Qing Li[1,*]

[1]State Key Laboratory of Cancer Biology, Department of Pathology; Xijing Hospital, Fourth Military Medical University, Shaanxi, 710032, China

[2]State Key Laboratory of Molecular Biology, Shanghai Key laboratory of Molecular Andrology, Institute of Biochemistry and Cell Biology, Shanghai Institutes for Biological Sciences, Chinese Academy of Sciences, 320 Yueyang Road, Shanghai 200031

[3]Department of Health Statistics, Fourth Military Medical University, Shaanxi, 710032, China

[4]Company 13, Student Brigade, Fourth Military Medical University, Xi'an, 710032, China

[5]Department of Neurosurgery; Xijing Hospital, Fourth Military Medical University, Shaanxi, 710032, China

*Correspondence and requests for materials should be addressed to:

F.Z. (zhf1975@fmmu.edu.cn), or

W.L (linwei@fmmu.edu.cn) or

Q.L (liqing@fmmu.edu.cn).

Table S1. Prognostic relevance of IDH1, TP53 and KI67 in diffuse astrocytomas (WHO grade II)

Markers	Authors	N	Seq	IHC (*Cut-off)	Positive Rate (%)	Correlation with OS
IDH1	Thon *et al*.[1]	127	Yes		99/127(78.1%)	No
IDH1	Ahmadi *et al*.[2]	100	Yes		79/100(79%)	No
IDH1	Waqar *et al*.[3]	83	-	Yes	46/83(55%)	Yes
IDH1	Mukasa *et al*.[4]	29	Yes		17/29(59%)	No
IDH1	Li *et al*.[5]	188	Yes		139/188(73.9%)	No
IDH1	Iwadate.[6]	38	-	Yes	27/38(82%)	Yes
TP53	Kraus et al.[7]	38	Yes		17/38(44%)	No
TP53	Mukasa et al.[4]	29	Yes		13/29(45%)	No
TP53	Hilton *et al.*[8]	96		Yes (10%)	44/96(45.8%)	No
TP53	Ogura *et al*.[9]	19		Yes (10%)	14/19(74%)	No
KI67	Hilton *et al.*[8]	96		Yes (0.75%)	41/96(42.7%)	No
KI67	Schiffer *et al.*[10]	50		Yes (8%)	-	Yes
KI67	McKeever *et al.*[11]	50		Yes (2%)	28/50(56%)	Yes
KI67	Torp and Alsaker [12]	22		Yes (2.7%)	9/22(40.9%)	Yes
KI67	Lind-Landström et al.[13]	104		Yes (4.5%)	52/104(50%)	No

*Cut-off point for prognostic value of labeling index.

IHC: immunohistochemistry; OS: overall survival.

Table S2. Prognostic relevance of *IDH1/2* mutations in lower grade gliomas (WHO grade II and/or III)

Authors	N	Subtypes (n) of the total cases	Assays	Correlation with OS in the total cases	Correlation with OS in DA
Kim et al[14]	360	DA(174), OA(64), OG(122)	Seq	No	-
Brat et al[15]	278	DA(30),OA(41),OG(65),AA(65),AOA(33),AO(44)	Seq	Yes	-
Sabha at al[16]	108	DA(56),AA(17),OG(10),OA(9),AO(10),AOA(6)	Seq+IHC	Yes	No
Goze et al[17]	131	DA(25),OG(71),OA(35)	Seq+IHC	Yes	-
Dahlrot et al[18]	66	DA(39),OG/OA(20),other(7)	IHC	Yes	-
Okita et al[19]	72	DA(49),OG(4),OA(19)	Seq+IHC	Yes	-
Yan et al[20]	445	DA(30),AA(52),OG(51),AO(36),other(276)	Seq	Yes in AA	No
Sanson et al[21]	221	DA(12),AA(18),OG(54),OA(34),AO(49),AOA(54)	Seq	Yes	-
Lewandowska et al[22]	139	DA(75),AA(17),OG(2),OA(24),other(21)	Seq+IHC	-	Yes
Zhang et al[23]	203	AA(56),AOA(103),AO(44)	Seq	Yes	-
Hartmann et al[24]	89	DA(40),OA(23),OG(26)	Seq	Yes	No
Chan et al[25]	214	DA(86),OA(38),OG(18),AA(63),AOA(3),AO(6)	Seq	Yes	-

DA, diffuse astrocytoma; OA, oligoastrocytoma; OG, oligodendroglioma; AA, anaplastic astrocytoma; AOA, anaplastic oligoastrocytoma; AO, anaplastic oligodendroglioma; Seq: sequencing; IHC : immunohistochemistry;OS, overall survival

References

1. Thon, N. *et al.* IDH1 mutations in grade II astrocytomas are associated with unfavorable progression-free survival and prolonged postrecurrence survival. *Cancer* **118**, 452-460 (2012).
2. Ahmadi, R. *et al.* No prognostic value of IDH1 mutations in a series of 100 WHO grade II astrocytomas. *J Neurooncol* **109**, 15-22 (2012).
3. Waqar, M. *et al.* Prognostic Factors in Lobar World Health Organization Grade II Astrocytomas. *World Neurosurg* **84**, 154-162 (2015).
4. Mukasa, A. *et al.* Significance of IDH mutations varies with tumor histology, grade, and genetics in Japanese glioma patients. *Cancer Sci* **103**, 587-592 (2012).

5. Li, M.Y. *et al.* Isocitrate dehydrogenase 1 Gene Mutation Is Associated with Prognosis in Clinical Low-Grade Gliomas. *PLoS One* **10**, e0130872 (2015).
6. Iwadate, Y. *et al.* IDH1 mutation is prognostic for diffuse astrocytoma but not low-grade oligodendrogliomas in patients not treated with early radiotherapy. *J Neurooncol* **124**, 493-500 (2015).
7. Kraus, J.A. *et al.* TP53 alterations and clinical outcome in low grade astrocytomas. *Genes Chromosomes Cancer* **10**, 143-149 (1994).
8. Hilton, D.A., Love, S., Barber, R., Ellison, D. & Sandeman, D.R. Accumulation of p53 and Ki-67 expression do not predict survival in patients with fibrillary astrocytomas or the response of these tumors to radiotherapy. *Neurosurgery* **42**, 724-729 (1998).
9. Ogura, R. *et al.* Immunohistochemical profiles of IDH1, MGMT and P53: Practical significance for prognostication of patients with diffuse gliomas. *Neuropathology* **35**, 324-335 (2015).
10. Schiffer, D., Cavalla, P., Chio, A., Richiardi, P. & Giordana, M.T. Proliferative activity and prognosis of low-grade astrocytomas. *J Neurooncol* **34**, 31-35 (1997).
11. McKeever, P.E., Strawderman, M.S., Yamini, B., Mikhail, A.A. & Blaivas, M. MIB-1 proliferation index predicts survival among patients with grade II astrocytoma. *J Neuropathol Exp Neurol* **57**, 931-936 (1998).
12. Torp, S.H. & Alsaker, M. Ki-67 immunoreactivity, basic fibroblastic growth factor (bFGF) expression, and microvessel density as supplementary prognostic tools in low-grade astrocytomas. An immunohistochemical study with special reference to the reliability of different Ki-67 antibodies. *Pathol Res Pract* **198**, 261-265 (2002).
13. Lind-Landstrom, T., Habberstad, A.H., Sundstrom, S. & Torp, S.H. Prognostic value of histological features in diffuse astrocytomas WHO grade II. *Int J Clin Exp Pathol* **5**, 152-158 (2012).
14. Kim, Y.H. *et al.* Molecular classification of low-grade diffuse gliomas. *Am J Pathol* **177**, 2708-2714 (2010).
15. Brat, D.J. *et al.* Comprehensive, Integrative Genomic Analysis of Diffuse Lower-Grade Gliomas. *N Engl J Med* **372**, 2481-2498 (2015).
16. Sabha, N. *et al.* Analysis of IDH mutation, 1p/19q deletion, and PTEN loss delineates prognosis in clinical low-grade diffuse gliomas. *Neuro Oncol* **16**, 914-923 (2014).
17. Goze, C. *et al.* Imaging growth and isocitrate dehydrogenase 1 mutation are independent predictors for diffuse low-grade gliomas. *Neuro Oncol* **16**, 1100-1109 (2014).
18. Dahlrot, R.H., Kristensen, B.W., Hjelmborg, J., Herrstedt, J. & Hansen, S. A population-based study of low-grade gliomas and mutated isocitrate dehydrogenase 1 (IDH1). *J Neurooncol* **114**, 309-317 (2013).
19. Okita, Y. *et al.* IDH1/2 mutation is a prognostic marker for survival and predicts response to chemotherapy for grade II gliomas concomitantly treated with radiation therapy. *Int J Oncol* **41**, 1325-1336 (2012).
20. Yan, H. *et al.* IDH1 and IDH2 mutations in gliomas. *N Engl J Med* **360**, 765-773 (2009).
21. Sanson, M. *et al.* Isocitrate dehydrogenase 1 codon 132 mutation is an important prognostic biomarker in gliomas. *J Clin Oncol* **27**, 4150-4154 (2009).

22. Lewandowska, M.A. *et al.* An analysis of the prognostic value of IDH1 (isocitrate dehydrogenase 1) mutation in Polish glioma patients. *Mol Diagn Ther* **18**, 45-53 (2014).
23. Zhang, C.B. *et al.* Correlation of IDH1/2 mutation with clinicopathologic factors and prognosis in anaplastic gliomas: a report of 203 patients from China. *J Cancer Res Clin Oncol* **140**, 45-51 (2014).
24. Hartmann, C. *et al.* Molecular markers in low-grade gliomas: predictive or prognostic? *Clin Cancer Res* **17**, 4588-4599 (2011).
25. Chan, A.K. *et al.* Combination genetic signature stratifies lower-grade gliomas better than histological grade. *Oncotarget* **6**, 20885-20901 (2015).
